# Prognostic Histopathological and Molecular Markers on Prostate Cancer Needle-Biopsies: A Review

**DOI:** 10.1155/2014/341324

**Published:** 2014-08-27

**Authors:** A. Marije Hoogland, Charlotte F. Kweldam, Geert J. L. H. van Leenders

**Affiliations:** Department of Pathology, Josephine Nefkens Institute, Room Be-206, Erasmus Medical Center, P.O. Box 2040, 3000 CA Rotterdam, The Netherlands

## Abstract

Prostate cancer is diverse in clinical presentation, histopathological tumor growth patterns, and survival. Therefore, individual assessment of a tumor's aggressive potential is crucial for clinical decision-making in men with prostate cancer. To date a large number of prognostic markers for prostate cancer have been described, most of them based on radical prostatectomy specimens. However, in order to affect clinical decision-making, validation of respective markers in pretreatment diagnostic needle-biopsies is essential. Here, we discuss established and promising histopathological and molecular parameters in diagnostic needle-biopsies.

## 1. Introduction

Prostate cancer is the most common cancer in Western men. Although prostate cancer follows an aggressive course in a significant number of men, most tumors do not cause significant clinical symptoms. Therefore individual assessment of a tumor's aggressive potential is crucial for clinical decision-making in men with prostate cancer.

In general, prostate cancer is diagnosed on needle-biopsies prompted by elevated serum prostate specific antigen (PSA) levels, suspicious digital rectal examination or trans-rectal ultrasonography findings, and/or clinical symptoms of urinary tract obstruction. In addition to elevated PSA levels and clinicoradiological signs of either local extension or metastasis, detailed histopathological characterization of prostate cancer at needle-biopsies predicts clinical tumor behavior and sustains therapeutic decision-making. In daily practice, the pathology report of prostate cancer includes the grade of differentiation according to the modified Gleason grading system, the number of biopsies infiltrated by prostate cancer, and a quantitative assessment of the tumor volume per biopsy in either length in mm or percentage of tumor [1, 2]. Implementation of novel histopathological and molecular markers is required for several reasons. While only half of the patients has a potentially life-threatening prostate cancer (Gleason score ≥ 7), 55–90% of patients with low-risk disease still undergo radical prostatectomy [3]. Active surveillance has become a widely used alternative for treatment after prostate cancer diagnosis. However, up to 33% of patients on active surveillance need therapeutic intervention after a median follow-up of 1.2–3.5 years [4–8]. Therefore, better stratification of prostate cancer patients with respect to clinical decision-making is necessary, especially in the predominant group of low- to intermediate-risk prostate cancers.

Another reason to implement novel markers in prostate cancer diagnosis and clinical decision-making is the considerable interobserver variability in Gleason grading among pathologists. This interobserver variability is particularly of significance in the large group of low- to intermediate-risk prostate cancer, as it can influence therapeutic approaches [8–10]. Contemporary modified Gleason grading in needle-biopsies demands adding the most common and highest Gleason grade to the final Gleason score, regardless of the amount of “highest” Gleason grade. In practice, when only a few atypical glands considered Gleason grade 4 are present together with a large volume of Gleason grade 3, the Gleason score is 7 and excludes patients from active surveillance in our institute. As considerable interobserver variability exists between the distinction of Gleason grades 3 and 4, important treatment decisions depend too much on individual pathologist's opinions. Thus, it is important to improve the reproducibility of Gleason grading by more objective parameters. In particular, molecular markers reflecting tumor biology could act as novel threshold in active surveillance or watchful waiting.

Finally, spatial heterogeneity of prostate cancer might lead to under- or, rarely, overestimation of prostate cancer aggressive potential on diagnostic needle-biopsies. In general, prostate biopsies only sample 0.05% to 0.5% of the total prostate volume, which might result in undersampling of the most significant area of prostate cancer tissue. In addition to improved image-guided needle-biopsy procedures, implementation of novel molecular markers might predict the presence of unsampled significant areas in case molecular aberrations precede pathologically discernible patterns.

Last decade, much effort has been put in the identification of novel histopathological and molecular markers to further improve prediction of tumor behavior in prostate cancer patients. The vast majority of research has focused on correlation of novel markers with static clinicopathologic parameters at radical prostatectomy such as Gleason score, pT-stage and surgical margin status, or biochemical recurrence after operation. However, in order to affect clinical decision-making, validation of respective markers in pretreatment diagnostic needle-biopsies is essential. Here, we discuss established and promising histopathological and molecular tissue markers in diagnostic needle-biopsies.

## 2. Pathologic Markers

### 2.1. Gleason Grading

The contemporary system for grading prostate cancer was developed by Gleason in the 1960s [11]. The Gleason grading system is solely based on the tumor's architecture. The Gleason score equals the sum of the two most common Gleason grades in radical prostatectomy, and the sum of the most common and highest Gleason grades in needle-biopsies. Up to date, the Gleason score is a strong predictor for disease progression, and one of the most important parameters in therapeutic decision-making.

In 2005, the Gleason grading system was modified at the International Society of Urological Pathology (ISUP) conference [1]. As a result, several tumor growth patterns classically considered as Gleason grade 3 were redefined as Gleason grade 4 [1]. Shortly after, small cribriform and glomeruloid glands have been reconsidered Gleason grade 4 as well [12–14]. As a result of this stage migration, Gleason score 7 has become the most common assigned score on prostatic needle-biopsies [15–17]. For instance, Helpap and Egevad showed in 368 needle-biopsy cases a significant change in distribution of modified Gleason score: Gleason score 2–4 decreased from 2.7 to 0%, Gleason score 5 decreased from 2.7 to 0%, Gleason score 6 decreased from 48 to 22%, but Gleason score 7 increased from 26 to 68% [16]. Generally, modified Gleason grading has improved the predictive value of grading prostate cancer.

#### 2.1.1. Modified Gleason Grading: Up- and Downgrading

The overall concordance of Gleason score between prostate needle-biopsies and radical prostatectomies has improved from 58% in classic Gleason score to 72% in modified Gleason score [16]. Uemura et al. found a decrease in downgrading Gleason score in needle-biopsies in comparison to radical prostatectomy (15% versus 20%), although overall Gleason score concordance rates did not change significantly [18]. To date, the overall upgrading rate at radical prostatectomy using the modified Gleason score ranges between 26 and 50% [19–22]. For instance, a large study containing 7643 radical prostatectomies with corresponding needle-biopsies demonstrated that 36% of cases (1841/5071) were upgraded from a needle-biopsy Gleason scores 5-6 to a higher grade at radical prostatectomy [19]. In the same study 72% (1143/1577) had matching Gleason score 7 on biopsy and radical prostatectomy, and Gleason score 8 on biopsy showed a similar distribution for radical prostatectomy Gleason score 4 + 3 = 7, 8, and 9-10 [19]. In men with Gleason score 9-10 on needle-biopsy, 58% (69/119) had a similar Gleason score on radical prostatectomy [19]. Proposed predictors for upgrading are age, high preoperative PSA, larger tumor percentage per core, number of positive cores, presence of perineural invasion, absence of inflammation, and high prostate volume [19, 21]. On the other hand, predictors for downgrading from any biopsy Gleason score to a lower Gleason score on radical prostatectomy were low preoperative PSA, lower tumor percentage per core, and larger prostate volume on radical prostatectomy [19, 21]. Although these predictors for up- and downgrading all showed strong statistical significance, the effect on survival was still small. If in one biopsy session, multiple biopsies show differing Gleason scores, for instance, Gleason scores 4 + 3 in one biopsy and 3 + 3 in another biopsy, also referred to as presence of ComboGS, patients have lower odds of upgrading at time of radical prostatectomy and decreased risk of prostate cancer specific mortality [23, 24].

#### 2.1.2. Modified Gleason Grading: Correlation with Pathologic Features at Radical Prostatectomy

The relationship between Gleason score on needle-biopsy and pathological stage on radical prostatectomy has improved since the implementation of the modified Gleason score [15, 25]. For instance, 4315/5205 men (83%) with Gleason score 6 on biopsy had organ-confined disease (pT2) at radical prostatectomy, while increasing Gleason score on biopsy was strongly associated with extraprostatic extension (pT3a) and seminal vesicle invasion (pT3b) [25]. Although high pathologic stage is related to positive surgical margins at radical prostatectomy and biochemical recurrence, it should be mentioned that it is not associated with a uniformly poor prognosis [26–28].

#### 2.1.3. Modified Gleason Grading: Predicting Patient Outcome

Billis et al. studied the impact of needle-biopsy Gleason score modification on biochemical recurrence free survival. Here, the modified Gleason score was a better predictor for biochemical recurrence than classic Gleason score [15]. Subsequently, the predictive value of the modified Gleason score has been validated in other large cohorts [18, 25]. Uemura et al. showed that the Gleason score was strongly associated with biochemical recurrence, only when the modified Gleason score was applied [18]. Furthermore, in a large study (*n* = 7850) from the Johns Hopkins Hospital, Pierorazio et al. correlated biopsy Gleason score with biochemical recurrence. Here, 95% of the patients with needle-biopsy Gleason score 6 had no biochemical recurrence after 5 years of follow-up [25]. In men with Gleason score 3 + 4 = 7 and Gleason score 4 + 3 = 7 on needle-biopsy the 5 year biochemical recurrence free survival rates were 83% and 65%, respectively. Men with Gleason score 4 + 4 = 8 or 9-10 had the lowest 5 year biochemical recurrence free survival rates, 63% and 35% respectively. Tollefson et al. indicated that distant metastasis and disease-specific death are best estimated by a combination of Gleason score, perineural invasion, and Ki-67 expression [29].

Altogether, modified Gleason grading has generally improved the concordance between biopsy and radical prostatectomy Gleason score, associates better with pathologic parameters at radical prostatectomy, and is more predictive for biochemical recurrence as well as metastasis and disease-specific death.

### 2.2. Tumor Quantification

Currently, the number of positive core biopsies should routinely be mentioned in pathology reports. Additional measures of prostate cancer volume in needle-biopsies better predict disease outcome. Various parameters have been proposed as measure of tumor extent, for example, tumor percentage in single biopsies, tumor length in single biopsies (mm), and number of negative biopsies. These quantitative assignments are required for most clinical nomograms. For instance, the Steyerberg nomogram incorporates number of positive biopsies, total cancer length (mm), and total “normal” tissue length (mm) to predict indolent disease on radical prostatectomy [30]. In addition, most active surveillance protocols are delimitated by the number of positive biopsies and/or a measure of tumor extent per biopsy [31, 32].

One well-studied example of tumor extent is the percentage of cancer in single biopsies, and many studies have confirmed its prognostic value in biopsies followed by radical prostatectomy [19, 25, 33–37], by dose-escalated external beam radiotherapy [38], or by a combination with hormonal treatment [39]. The location of positive biopsies can additionally be of therapeutic value, for instance for consideration of nerve-sparing surgery. The majority of these studies agree on the predictive value of tumor extent for endpoints such as biochemical recurrence [19, 25, 35–38, 40], metastasis [36–38], and disease-specific death [36, 38]. However, despite its statistical significance, the effect of tumor percentage in biopsies on survival is mostly small. Furthermore, all of the above mentioned publications used different methods to determine the percentage of cancer and different cutoff values. For instance, Vance et al. divided the percentage of cancer in single biopsies into four quartiles (<2.5%, <10%, <25%, and ≥25%) [38], while Nelson et al. categorized it in to 0–10%, 11–59%, and 60–100% [36].

Another frequently assessed measure on prostate biopsies is the percentage of positive biopsy cores, defined as the total number of positive cores divided by the total number of biopsy cores obtained. Studies evaluating its prognostic value have validated the independent predictive value for biochemical recurrence free survival [35, 39, 41, 42]. In 2011 Huang et al. analyzed needle-biopsies of 1056 patients treated with external beam radiotherapy and/or hormonal therapy [43]. Using a cutoff value of >50% positive biopsy cores they found that percentage of positive biopsy cores is a powerful and independent predictor for distant metastasis free and overall survival [43]. However, when the percentage of positive biopsy cores was adjusted for percentage of cancer in needle-biopsies in another study, it did not provide any additional superior risk stratification for biochemical recurrence, distant metastasis, or disease-specific death [38].

While measuring tumor extent is generally straight-forward, minor controversies exist for instance for quantifying discontinuous prostate cancer foci in single biopsies. One could regard separate foci as being part of the same tumor and measure the distance between the outermost foci including intervening normal prostate tissue, or only measure malignant areas without intervening stroma. In this case, recent studies show that discontinuous foci of prostate cancer in needle-biopsies should be regarded and measured as one continuous lesion [44, 45].

In short, tumor volume is an important parameter for disease extent, but there is no consensus yet on the best methodology for its assessment. It is clear that assignment of the number of positive biopsies requires identification of separate biopsy cores, even when they are fragmented due to technical procedures. This can be performed by including only one needle-biopsy per cassette, or marking multiple individual cores in one cassette for instance by inking. It is advised that no more than 3 biopsies should be included in one cassette, provided that measures are taken to prevent their curling and floating [46]. The extent of cancer in individual cores is performed by actual measuring of the tumor's length with a ruler or by estimation of tumor percentage by eye-balling. Measuring tumor length is objective and exact, although more time-consuming. If estimation of percentage is applied in daily practice, one should take into account that detection of prostate cancer in short needle-biopsies due to suboptimal technical procedures can result in over-estimation of tumor percentage; for instance presence of 2 mm prostate cancer in a 5 mm biopsy results in a tumor percentage of 40%, which might erroneously exclude patients from active surveillance.

### 2.3. Perineural Invasion

The significance of perineural invasion in prostate cancer biopsies remains questionable. In a systematic review Harnden et al. addressed important limitations of 21 studies on perineural invasion in biopsies followed by radical prostatectomy or radiation therapy [47]. First, the presence of nerves in biopsies was not mandatory for patient inclusion. Second, the number of biopsy cores taken and the number of nerves present ranged widely. Third, pathologists were not obligated to routinely report on presence of perineural invasion; there were striking differences in the frequency of perineural invasion when biopsies were reviewed for study purpose. Interestingly, only 43% (18/42) of surveyed urologists think that presence of perineural invasion on prostate biopsy should influence treatment [48]. Ten out of 18 surveyed urologists (56%) indicated that it helps planning nerve-sparing surgery. In contrast, nerve-sparing surgery was considered as a confounding factor in the studies mentioned by Harnden et al. [47]. However, despite limitations Harden et al. conclude that the weight of evidence in studies ascribing prognostic significance to perineural invasion appears to suggest that perineural invasion should influence clinical decision-making [47]. For instance, Quinn et al. demonstrated in a large cohort (*n* = 696) that perineural invasion was a significant predictor for outcome in a multivariable analysis [49]. Delancey et al. demonstrate that perineural invasion on prostatic needle-biopsy is an independent predictor for biochemical recurrence, disease-specific survival, and overall survival after radical prostatectomy [50].

### 2.4. Intraductal Carcinoma: A High-Risk Lesion

**Figure 1:**
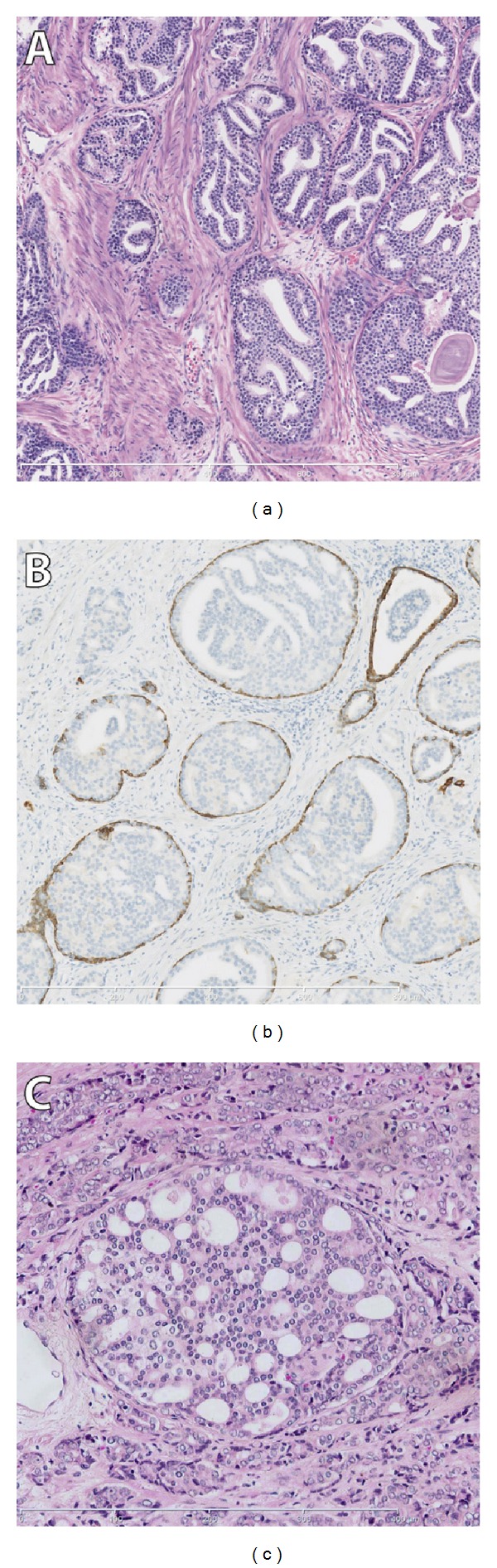
(a) intraductal carcinoma of the prostate (100x magnification). (b) 34BE12 immunohistochemistry, demonstrating the presence of basal cells supportive for intraductal carcinoma (100x magnification). (c) Cribriform growth pattern of Gleason grade 4 adenocarcinoma (200x magnification).

Intraductal carcinoma of the prostate is defined as a well-circumscribed lesion surrounded by an intact basal cell layer distended by overtly malignant-appearing epithelial populations [51] (Figures 1(a) and 1(b)). Intraductal carcinoma forms a morphologic continuum with high-grade prostate intraepithelial neoplasia (PIN), which is the generally accepted precursor of prostate cancer. While PIN is recognized by the presence of cytologically malignant cells in preexistent prostate glands of normal architecture, these glands should be enlarged in intraductal carcinoma. In contrast to PIN, which can be an isolated finding on prostate biopsy, intraductal carcinoma is associated in 90% of cases with invasive carcinoma, mostly of intermediate or high risk [52]. Up to date, three studies on intraductal carcinoma all demonstrated its independent predictive value for biochemical recurrence after radical prostatectomy, treatment failure, and distant metastasis in biopsies [53–55]. van der Kwast et al. showed that intraductal carcinoma on needle-biopsies prior to external beam radiotherapy with or without androgen deprivation therapy strongly predicted biochemical recurrence and early distant metastasis in a cohort of 118 intermediate and 132 high risk prostate cancer patients [55].

### 2.5. Percentage Gleason Grades 4 and 5

A proposed adaption to the Gleason grading system by Vis et al. is the reporting of percentage of Gleason grades 4/5 in prostatic needle-biopsies [37]. In this study, containing 281 patients, amount of high-grade cancer (length in mm, or percentage) in the core biopsy was an independent prognostic factor for biochemical recurrence and clinical relapse of prostate cancer [37]. In addition, when biopsy Gleason score 7 cancers were subcategorized into 3 + 4 and 4 + 3 cancers, the amount of high-grade cancer was the strongest predictor of biochemical recurrence free survival, whereas the Gleason grading system was rejected in the presence of high-grade components [37]. So far, no other studies have yet validated the predictive value of this parameter on prostatic needle-biopsies.

### 2.6. Gleason Grade 4 Patterns: Individual Prognostic Value

Gleason grade 4 tumors represent a diverse group, containing at least 4 distinctive growth patterns: fused, cribriform, ill-defined, and glomeruloid [1, 14, 56]. Recently, Dong et al. studied the prognostic value of these distinct Gleason grade 4 patterns and showed that cribriform growth, in particular, was strongly associated with biochemical recurrence and distant metastasis after radical prostatectomy [57] (Figure 1(c)). Only one publication has yet demonstrated the adverse prognostic value of cribriform growth pattern in a biopsy series, with radiotherapy as treatment, and biochemical recurrence as clinical endpoint [54].

### 2.7. Extraprostatic Extension

Rarely, prostate cancer is infiltrating extraprostatic fat tissue, seminal vesicle, or ejaculatory ducts on needle-biopsies. Fat invasion by tumor on prostatic needle-biopsy is considered as extraprostatic extension by 81% of surveyed pathologists from the European Network of Uropathology [58]. If present, these features should be mentioned in diagnostic needle-biopsies. Since signs of extraprostatic extension are mostly seen in voluminous prostate cancer with high Gleason score, these patients could be less eligible for radical treatment.

### 2.8. Tumor-Associated Macrophages

Solid tumors grow in a complex and dynamic stroma involving various cell types, for example, leukocytes, fibroblasts, and endothelial cells. Tumor-associated macrophages are part of the tumor microenvironment and seem to influence solid tumor progression, for example, in colon, breast, and ovarian cancer [59]. Nonomura et al. were the first to study its prognostic relevance in prostate biopsies [60]. The presence of tumor-associated macrophages, as immunohistochemically identified by the CD68 antibody in a cohort of 71 prostate cancer patients treated with hormones, was associated with disease recurrence after hormonal therapy. Furthermore, tumor-associated macrophages count (>22 at 400x magnification), PSA level, Gleason score ≥7, and extracapsular extension were independent predictors for biochemical recurrence free survival in multivariate analysis [60]. Studies on tumor-infiltrating lymphocytes in prostatic needle-biopsies have not yet been performed.

### 2.9. Summary

- To date, Gleason grading on prostatic biopsy is the most important predictor for biochemical recurrence, distant metastasis, and cancer-specific mortality in prostate cancer.
- Despite the modification in 2005 by the ISUP, Gleason score upgrading at radical prostatectomy still ranges between 26 and 50%.
- Proposed predictors of upgrading are high preoperative PSA, larger tumor percentage per biopsy core, number of positive cores, and presence of perineural invasion.
- Proposed predictors of downgrading are smaller tumor percentage per core and large prostate volume.
- Differing Gleason scores on prostate needle biopsies could be a useful tool in decreasing the odds of upgrading.
- Tumor quantification is an important prognostic factor and implemented in clinical nomograms of prostate cancer; there is, however, no consensus on the best methodology for its assessment. Furthermore, despite its fundamental role in clinical nomograms, the effect on survival is relatively small.
- Presence of perineural invasion on needle-biopsies is an independent predictor for survival, and therefore a recommended parameter to add in standardized pathology reporting.
- The amount of Gleason grade 4/5 in needle-biopsies seems to have prognostic value; however, it needs to be validated in large cohorts with strong endpoints.
- Cribriform growth and/or intraductal spread are promising histopathological markers in needle-biopsies.
- The prognostic value of tumor-associated inflammation has recently been acknowledged in multiple solid tumors and needs to be further investigated in prostate cancer.

## 3. Molecular Markers

### 3.1. Ki-67

The Ki-67 protein is a cell proliferation marker, which is expressed in G_1_, S, G_2_, and M phases of the cell cycle being absent in resting (G_0_) cells. The Ki-67 labeling index as determined by the MIB-1 antibody is the best studied prostate cancer marker in needle-biopsies up to date [61–66]. Ki-67 labeling index shows a strong correlation with Gleason score on diagnostic biopsies [61, 63], on subsequent radical prostatectomy [64–66], or both [62]. In a cohort of 91 patients, Rubio et al. found Ki-67 (cut-off value of 5% positive nuclei) on needle-biopsies to be a marker for disease-free survival after radical prostatectomy in univariate analysis [62]. Zellweger and colleagues found that Ki-67 labeling index of ≥10% at biopsy cores in 279 patients independently predicted seminal vesicle invasion and Gleason score on subsequent radical prostatectomy [66]. In addition, they found that Ki-67 was the only independent marker for postoperative biochemical recurrence in a subgroup of low-volume (<7%) or low-grade (Gleason score < 7) prostate cancer at needle-biopsies. Tolonen et al. demonstrated that Ki-67 in 247 primarily endocrine-treated prostate cancer patients was associated with progression-free survival [63]. Ki-67 labeling ≥ 10% on 293 microarrayed needle-biopsies had independent predictive value for disease-specific death together with Gleason score and PSA [61]. Recently, Tollefson et al. calculated in a cohort of 451 prostate cancer needle-biopsies that every 1% increase in Ki-67 expression resulted in a 12% increased risk of cancer-specific death after radical prostatectomy [29]. Whereas these studies all show additional value of Ki-67 expression in needle-biopsies with aggressive disease features, two studies focused on the relation of Ki-67 labeling in needle-biopsies and presence of indolent disease on radical prostatectomy. In a well-defined screening cohort Vis et al. were not able to find a significant association of Ki-67 ≥ 10% with significant prostate cancer at radical prostatectomy in 81 patients [64]. Wolters et al. were also not able to find a significant association of high Ki-67 expression (>3%) with significant prostate cancer at radical prostatectomy in 86 patients [65]. Taken together, enhanced Ki-67 labeling at needle-biopsy is associated with adverse clinicopathologic features and disease-specific death in general prostate cancer populations.

### 3.2. p27

p27^kip1^ is a cyclin-dependent kinase (cdk) inhibitor. It inhibits cell cycle progression in G_1_ phase by preventing activation of cyclin E-cdk2 and cyclin D/-cdk4 complexes [67]. Loss of p27 has been widely associated with progression of different tumor types, including breast, colorectal, and lung cancer [67]. Generally, p27 expression in prostate cancer needle-biopsies correlates well with the p27 labeling in radical prostatectomy samples [64, 67]. In prostate cancer, various groups have shown that loss of p27 expression was associated with more aggressive disease parameters. Thomas et al. demonstrated that low expression (<30%) of p27 in needle-biopsies correlated with higher Gleason score and pT-stage at radical prostatectomy [67]. In this relatively small cohort of 44 patients, tumors with low p27 expression showed a trend towards shortened biochemical recurrence free survival after operation. Vis et al. showed that p27 expression in <50% together with Gleason score were the only significant parameters to predict clinically significant disease at radical prostatectomy in a screen-detected cohort of 81 prostate cancer patients [64]. In addition, we found that p27 in <90% in a low-risk prostate cancer cohort was an independent parameter of clinically significant prostate cancer in 86 radical prostatectomy samples [65]. Therefore, loss of p27 is a marker of more aggressive prostate cancer, although the number of patients is limited and different standard cutoff levels have been used by various groups.

### 3.3. EZH2

Enhancer of zeste homologue 2 (EZH2) belongs to the Polycomb-group proteins and is important in maintaining cell identity and regulation of the cell cycle [68, 69]. EZH2 has been reported to be of both prognostic and therapeutic value in different tumors, such as small cell lung carcinoma [70], breast cancer [71, 72], cervical carcinomas [73], urinary tract carcinoma [74], and lymphoma [75]. Through gene expression profiling, EZH2 was found to be overexpressed in hormone-refractory metastatic prostate cancer [69]. Overexpression of EZH2 in radical prostatectomy samples was associated with poor prognosis [69, 76–78]. In a set of 86 needle-biopsies of screen-detected low-risk prostate cancer, EZH2 expression >1% was associated with clinically significant tumors on radical prostatectomy, defined as presence of extraprostatic extension, Gleason grade 4/5 or tumor volume ≥0.5 mL [65]. In the same study, no prognostic value was found for Polycomb-group protein BMI1. Tolonen et al. found independent predictive value for EZH2 (expression level of >15%) for progression-free survival in 247 hormone-treated biopsies [63]. While enhanced EZH2 expression in prostate cancer biopsies has independent prognostic value, there is no consensus yet on cutoff points in clinical practice.

### 3.4. TMPRSS2:ERG

Fusion of the androgen-dependent* TMPRSS2* gene to ETS-transcription factor* ERG* (*TMPRSS2:ERG*) is one of the most common genetic alterations in prostate cancer occurring in 50%–70% of tumors [79]. Many groups have analyzed the presence of* TMPRSS2:ERG* fusion or ERG protein expression in prostate cancer cohorts with variable outcome [80–92]. Barros-Silva et al. used fluorescent in situ hybridization (FISH) to detect* TMPRSS2-ERG* rearrangement in a cohort of 200 biopsies and found an association with low PSA levels at diagnosis and low Gleason score [93]. In needle-biopsies immunohistochemical ERG detection can be used to discriminate prostate cancer from its mimickers, although the additional value to other markers such as p63, basal cell keratin 5, and AMACR is limited [92, 94–99]. In an active surveillance cohort of 265 men, Berg et al. found a strong correlation between ERG protein expression and disease-progression [100]. Likewise, Hagglof et al. showed a shorter survival of prostate cancer patients on watchful waiting when ERG was expressed [101]. They found a cumulative 2-year progression rate of 59% in the ERG-positive group versus 22% in the ERG-negative group. Finally, expression of ERG in high-grade PIN was associated with a higher chance of developing prostate cancer at subsequent biopsies [102]. ERG immunohistochemistry is an easy to perform methodology for detecting TMPRSS2:ERG fusion in prostate cancer. While the clinical relevance of TMPRSS2:ERG fusion on radical prostatectomy specimens is unresolved yet, most reports indicate that ERG expression on biopsy, in surveillance cohorts can select a subgroup with higher chance to progression.

### 3.5. Neuroendocrine Differentiation

In many prostate cancers, scattered tumor cells show neuroendocrine differentiation as demonstrated by antibodies to Chromogranine, Synaptophysine, or Serotonine. After hormonal therapy, the relative number of neuroendocrine cells is increased, putatively due to their androgen-independent nature [103–105]. Despite extensive studies on neuroendocrine differentiation in relation to castration-resistance, this feature is rarely studied in pretreatment biopsies. Krauss and colleagues have shown that Chromogranine An expression of >1% in prostate cancer biopsies is an independent predictor for distant metastasis and cause-specific survival after primary radiation therapy [106, 107].

### 3.6. c-MYC

The oncogene* c-MYC* located at 8q24 encodes a transcription factor involved in cell cycle progression, cell growth, proliferation, protein synthesis, mitochondrial function, stem cell renewal, and DNA replication [108, 109].* c-MYC* is amplified in approximately 70% of clinical prostate cancer [93, 110, 111]. Ribeiro et al. found that patients with gain of* MYC* gene copy numbers in a group of 60 prostate cancer needle-biopsies using FISH were significantly at risk for disease-specific death [110]. Bastacky et al. showed that amplification of* c-MYC* in needle-biopsies with high-grade PIN was predictive of finding prostate cancer in subsequent biopsies [112]. The potential predictive value of* c-MYC* was confirmed by Zafarana et al. in a cohort of 126 needle biopsies, where they found* c-MYC* gain alone to be prognostic for tumor recurrence after radiotherapy [111].* c-MYC* gain combined with loss of* PTEN* further increased the predictive value for recurrence after radiotherapy.

### 3.7. PTEN

*Phosphatase and tensin homologue* (*PTEN*) is a tumor suppressor gene which is inactivated in many different tumors, including prostate cancer [113]. On large cohorts of radical prostatectomy samples and transurethral resection (TUR) samples,* PTEN* loss has been associated with bone metastases, resistance to radiotherapy and chemotherapy, progression to androgen-independent disease, and disease recurrence after surgery [113, 114]. Zafarana et al. found that* PTEN* loss alone and in combination with* c-MYC* gain were independently associated with biochemical recurrence after radiation therapy in a group of 126 intermediate-risk prostate cancer biopsies [111].

### 3.8. APC

Using quantitative methylation-specific PCR (QMSP) Henrique and colleagues showed that hypermethylation of* APC*,* GSTP1*, and* RASSF1A* in 83 prostate cancers at sextant needle-biopsies was associated with poor disease-specific survival [115]. Besides clinical stage, hypermethylation of* APC* was independently predictive for decreased disease-free and disease-specific survival. Methylation of* CCND2* and* RARB2* in the same study did not have predictive value for disease outcome.

### 3.9. Molecular Signatures

Subgroups with unique molecular, pathologic, clinical, and therapy-sensitivity, as have been defined in breast cancer, have not been delineated in prostate cancer yet. Nevertheless, recently gene signatures have been put forth to predict prostate cancer behavior. Klein et al. demonstrated that a 17-gene assay was able to identify patients with high-grade and high-stage disease at radical prostatectomy in a cohort of 395 men with low- to intermediate-risk prostate cancer at biopsies [116]. In addition, Irshad et al. identified a 3-gene signature of* FGFR1*,* PMP22*, and* CDKN1A*, which could accurately predict the outcome of low Gleason score prostate cancer in different cohorts [117]. For clinical implementation and validation this group applied immunohistochemistry for the respective proteins. In a limited matched cohort of 43 low-risk prostate cancer patients on active surveillance they were perfectly able to identify patients with failure upon active surveillance by reduced expression of these 3 proteins. Gene-based signatures therefore are a promising tool for risk stratification and might gain wider application if translation to easy-to-use procedures such as immunohistochemistry is available.

### 3.10. Biopsy Markers without Prognostic Value

While numerous markers have been shown to correlate with adverse clinicopathologic parameters on radical prostatectomy, just a limited number of these has been investigated in pretreatment needle-biopsies. Briefly, we also want to mention markers that have been investigated on biopsies but did not show additional value. In a group of 91 prostate cancer needle-biopsies, Bax, Bcl-2, and Cox-2 did not show independent predictive value for disease-free survival, although Cox-2 was predictive in univariate analysis [62]. On a large cohort of 247 patients with primary endocrine treatment, Tolonen et al. demonstrated that minichromosome maintenance protein 7 (MCM7) was a significant albeit not independent marker for disease-progression [63]. We validated the prognostic value of Cystein-rich secretory protein 3 (CRISP-3) and *β*-Microseminoprotein (*β*-MSP) in a screening cohort of 174 men. We found that expression of these markers was correlated with Gleason score, tumor volume, and pT-stage and significant disease on subsequent radical prostatectomy samples but were not able to predict recurrence [118].

### 3.11. Summary

- The cell proliferation marker Ki-67 (MIB-1) is the best studied immunohistochemical marker in prostate with independent prognostic value in multiple studies.
- Cyclin-dependent kinase inhibitor p27 and Polycomb-group protein EZH2 are both promising immunohistochemical markers for predicting disease outcome.
- The clinical significance of* TMPRSS:ERG* fusion or ERG protein overexpression is still controversial, although some studies demonstrate adverse prognostic value in active surveillance/watchful waiting cohorts.
- Amplification of* c-MYC*,* PTEN* loss, and* APC* hypermethylation are promising markers for predicting disease-specific death, albeit only demonstrated in a small number of biopsy cohorts.
- Identification of complex gene signatures offers novel promising platforms for predicting disease-outcome. Routine implementation in local pathology laboratories is currently not applicable.

## 4. Conclusions

Investigation of potential novel predictive markers in prostate cancer needle-biopsies is of importance to affect clinical decision-making and to be implemented in daily practice. A prerequisite in analyzing novel markers on needle-biopsies is the presence of well-characterized patient cohorts with clinical follow-up and availability of prostate cancer tissue for actual testing. Secondary and tertiary cancer centers often do not have original tissue blocks for further research on site. In addition, small foci of prostate cancer are often not present anymore in the remaining paraffin block. Incorporation of both detailed histopathological prostate cancer features and molecular markers can support optimal therapeutic decision-making in individual patients.

Comprehensive reporting of novel histopathological parameters such as percentage Gleason grade 4/5, intraductal carcinoma and potentially Gleason grade 4 growth pattern is a fast and cheap way to better estimate a prostate cancer's future clinical behavior in daily practice. Various molecular markers such as Ki-67, p27, EZH2, and ERG immunohistochemistry, as well as* c-MYC* and* PTEN* in situ hybridization, can putatively sustain and improve pathologic diagnosis. The variability in patient cohorts, clinical endpoints, technical methodology, and quantification, however, require prospective studies in well-characterized patient groups before implementation in daily practice is feasible. Identification of complex gene-signatures is a recent and promising tool in stratification of prostate cancer patients, though still costly and not easily applicable in daily practice.

Last decade, the diagnosis of prostate cancer has changed significantly. Pathologic Gleason scoring has been modified; sextant biopsies have widely been replaced by 10 to 12 or more biopsy sampling protocols; image-guided biopsies supplement established biopsy schemes and facilitate sampling of biologically relevant tumor areas. It is difficult to interpret the additional value of established prognostic factors of earlier studies in contemporary prostate cancer health care. We advocate that incorporation of novel histopathological parameters such as percentage Gleason grade 4/5, presence of intraductal carcinoma, and Gleason grade 4 growth patterns in daily pathology practice as well as prostate cancer studies offer an inexpensive and short-term opportunity to improve prostate cancer health care and interpret the additional value of promising molecular markers.
